# Imaging Lymph Nodes with Nanoparticles

**DOI:** 10.1371/journal.pmed.0010067

**Published:** 2004-12-28

Accurate staging of cancers is one of the most important parts of the work up of patients for both prediction of prognosis and determination of the most appropriate treatment. And an essential part of this work up is assessing whether or not there has been lymphatic spread. Current methods include surgical removal of nodes for examination and various types of imaging, ranging from ultrasound to newer technologies such as magnetic resonance imaging (MRI). All these methods have problems; some are very invasive, others are very time consuming, and none are completely reliable.[Fig pmed-0010067-g001]

**Figure pmed-0010067-g001:**
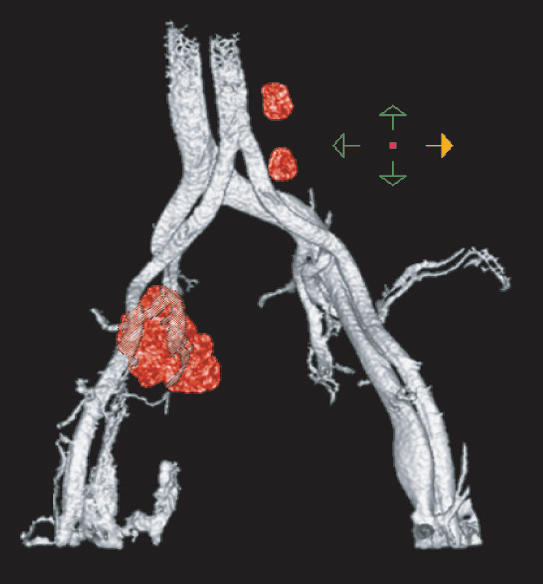
3-D image of lymph node after automated analysis

However in one of the more exciting crossovers from chemistry into medicine, researchers have developed nanoparticles to improve the diagnostic accuracy of MRI. The nanoparticles contain a central superparamagnetic iron oxide core and are covered by dextran, imparting long circulation times and biocompatibility. When injected intravenously, the nanoparticles localize to lymphoid tissue, and are internalized into macrophages. There is then a decrease in signal intensity on T2- and T2*-weighted images, and when metastases are present there is a recognizably abnormal pattern on MRI scans.

In a previous paper published in the *New England Journal of Medicine*, Ralph Weissleder and colleagues described using these nanoparticles to assess lymphoid spread in patients with prostate cancer. Now, in a paper published in this month's *PLoS Medicine*, they have gone further by extending the analysis to patients with different types of cancer, and producing an algorithm that allows semiautomation of the procedure.

The authors developed the algorithm in a training group of 36 patients and then validated it in a group of 34 patients. The results are encouraging: the analysis showed a sensitivity of 98% (95% confidence interval, 88%–99%) and a specificity of 92% (95% confidence interval, 87%–96%). The advantages of automating this procedure are substantial, not least because it can remove the problem of different observers assessing data differently.

And what is more, once the data have been collected and assessed it is possible to reconstruct a virtual picture of the patient's lymph nodes, thus potentially allowing accurate surgical removal of the nodes.

